# Complete Genome Sequence of *Bacillus subtilis* Strain 29R7-12, a Piezophilic Bacterium Isolated from Coal-Bearing Sediment 2.4 Kilometers below the Seafloor

**DOI:** 10.1128/genomeA.01621-16

**Published:** 2017-02-23

**Authors:** Yuli Wei, Junwei Cao, Jiasong Fang, Chiaki Kato, Weicheng Cui

**Affiliations:** a Shanghai Engineering Research Center of Hadal Science and Technology, College of Marine Sciences, Shanghai Ocean University, Shanghai, China; b College of Natural and Computational Sciences, Hawaii Pacific University, Kaneohe, Hawaii, USA; c Department of Marine Biodiversity Research, Japan Agency for Marine-Earth Science and Technology, Yokosuka, Japan

## Abstract

Here, we report the genome sequence of *Bacillus subtilis* strain 29R7-12, a piezophilic bacterium isolated from coal-bearing sediment down to ~2.4 km below the ocean floor in the northwestern Pacific. The strain is a Gram-positive spore-forming bacterium, closely related to *Bacillus subtilis* within the phylum *Firmicutes*. This is the first complete genome sequence of a *Bacillus subtilis* strain from the deep biosphere. The genome sequence will provide a valuable resource for comparative studies of microorganisms from the surface and subsurface environments.

## GENOME ANNOUNCEMENT

Microbial life has been detected in marine sediments as deep as about 2.5 km below the seafloor (bsf) (1) and viable bacteria have been isolated from subsurface sediment down to 2.4 km bsf (J.F., C.K., unpublished data). However, we still do not know the physiology and metabolism of microorganisms in the deep biosphere, as the deep subsurface prokaryotic cells are mostly resistant to cultivation and <0.1% of all microscopically and/or molecular genetically detected cells have been isolated and characterized (2–4). In this study, we obtained the complete genomic sequence of *B. subtilis* 29R7-12, isolated from the deep subsurface ~2.4 km below the ocean floor. *B. subtilis* is a well-characterized model bacterium, endowed with complex regulatory and metabolic networks allowing them to thrive in a broad spectrum of environments (5–7). In addition, *B. subtilis* is a commensal bacterium able to form metabolically inactive dehydrated endospores allowing survival in nutrient-depleted and other extreme environments (8, 9). Here, we present the complete genome of *B. subtilis* 29R7-12.

*Bacillus subtilis* 29R7-12 was cultivated in 100 mL of marine broth 2216 at 45°C and 0.1 MPa for 24 h, and genomic DNA was extracted and purified using a Qiagen genomic-tip 500/G kit (QIAGEN, Düsseldorf, Germany) according to the manufacturer’s protocol. Whole-genome shotgun sequencing was carried out using PacBio (Pacific Biosciences, Menlo Park, CA) single-molecule-real-time (SMRT) sequencing technology at the Chinese National Human Genome Center in Shanghai, China. The genome was assembled *de novo* utilizing the PacBio Hierarchical Genome Assembly Process version 3 (HGAP3) (10). The complete genome sequence of* Bacillus subtilis* 29R comprises a 4,121,999 bp circular chromosome (with ~332-fold coverage and G+C content of 43.4%) and two plasmids (64,604 bp and 17,447 bp).

The genome was annotated using the NCBI Prokaryotic Genome Annotation Pipeline (http://www.ncbi.nlm.nih.gov/genome/annotation_prok/). A total of 4,187 protein-coding sequences were identified, as well as 10 (5S, 16S, and 23S) rRNA operons and 87 tRNA genes. Lee et al. showed that bacterial species with eight or more 16S rRNA genes account for 11% of the unique entries in the Ribosomal RNA Database (rrnDB), and they belong to the phylum *Firmicutes* or the class *Gammaproteobacteria* (11). *Bacillus subtilis* 29R7-12 contains more copies of rRNA operons than most microbial genomes. Klappenbach et al. showed that there is a positive correlation between the number of rRNA genes in an organism’s genome and the capacity of that organism to respond to favorable growth conditions (12). Therefore, we infer that the copy number of 16S rRNA genes in *Bacillus subtilis* 29R7-12 may correlate with the ecological strategy of resource utilization and the environmental fitness.

### Accession number(s).

The complete genome sequence of *Bacillus subtilis* strain 29R7-12 has been deposited in GenBank under accession no. CP017763 (chromosome), CP017764 (plasmid 1), and CP017765 (plasmid 2).
